# Family Strengths, Self-Esteem, and Depression in Older Adults with Kidney/Liver Disability: A Dyadic Analysis

**DOI:** 10.1093/geroni/igaf122.4119

**Published:** 2025-12-31

**Authors:** JaeWon Hyun, Bomgyeol Kim, Hun Kang, JiYeon Choi

**Affiliations:** Yonsei University, Seoul, Republic of Korea; Yonsei University College of Nursing, Seoul, Republic of Korea; Yonsei University, Seoul, Republic of Korea; Yonsei University, Seoul, Republic of Korea

## Abstract

Chronic kidney or liver disease often leads to disability status. As populations age, these challenges increase emotional, physical, and social strain on individuals and family caregivers. While family strengths reduce depressive symptoms, few studies have examined the underlying mechanisms within patient-caregiver dyads affected by these conditions. Using the data from the 2023 Disability and Life Dynamics Panel (DLDP), we examined how self-esteem mediate the relationship between family strengths and depressive symptoms in 161 chronic kidney disease dyads (adults aged ≥50 years undergoing dialysis or post-kidney transplant) and 76 chronic liver disease dyads (adults aged ≥50 years with chronic liver dysfunction). The Actor–Partner Interdependence Mediation Model showed good fit for both groups. In kidney disease dyads, patients’ and caregivers’ family strengths positively influenced their self-esteem (β = 0.28, p=.004; β = 0.38, p<.001), which in turn reduced their own depressive symptoms (β = −0.48, p<.001; β = −0.28, p=.002). Additionally, patients’ self-esteem was negatively associated with caregivers’ depressive symptoms (β = −0.18, p=.026). In liver disease dyads, patients’ family strengths increased self-esteem (β = 0.42, p=.010), decreasing their depressive symptoms (β = −0.58, p<.001; indirect effect β = −0.24, p=.041), while caregivers’ family strengths directly reduced their depressive symptoms (β = −0.25, p=.042). No significant partner effects were observed in liver dyads. Our findings highlight the importance of family strengths and self-esteem in mitigating depressive symptoms among older adults with chronic kidney or liver disease and their caregivers. These results suggest that enhancing family strengths and supporting self-esteem may be effective strategies to promote psychosocial well-being in this population.

